# From Biomass to Adsorbent: A Comprehensive Review on Bio-Derived Carbons for Dye Removal

**DOI:** 10.3390/polym18020180

**Published:** 2026-01-09

**Authors:** Buvaneswari Kuppusamy, Fathima Rigana Mohamed Ismail, Preethi Balakrishnan, Seong-Cheol Kim, Shakila Parveen Asrafali, Thirukumaran Periyasamy

**Affiliations:** 1 Department of Chemistry, KCG College of Technology, Karapakkam, Chennai-97 600097, Tamilnadu, India; 2 Department of Chemistry, Faculty of Engineering, Karpagam Academy of Higher Education, Coimbatore 641021, Tamilnadu, India; 3 School of Chemical Engineering, Yeungnam University, Gyeongsan 38541, Republic of Korea; 4 Department of Fiber System Engineering, Yeungnam University, Gyeongsan 38541, Republic of Korea

**Keywords:** bio-carbons, dye adsorption, sustainability, cost-effective and reusability

## Abstract

The escalating release of synthetic dyes from textile and allied industries has become a pressing global environmental issue due to their toxicity, persistence, and resistance to biodegradation. Among the various treatment strategies, adsorption has emerged as one of the most efficient, economical, and sustainable techniques for dye removal from aqueous environments. This review highlights recent advances in bio-derived adsorbents—particularly raw biomass powders, biochars, and activated carbons—developed from renewable waste sources such as agricultural residues, fruit peels, shells, and plant fibers. It systematically discusses adsorption mechanisms, the influence of process parameters, kinetic and thermodynamic models, and regeneration performance. Furthermore, the review emphasizes the superior adsorption efficiency and cost-effectiveness of biomass-derived carbons compared to conventional adsorbents. The integration of surface modification, magnetization, and nanocomposite formation has further enhanced dye uptake and reusability. Overall, this study underscores the potential of biomass-derived materials as sustainable alternatives for wastewater treatment and environmental remediation.

## 1. Introduction

Humanity is currently grappling with two significant global challenges: the energy crisis and environmental pollution. Rapid population growth, coupled with increasing water scarcity, has intensified the contamination of water bodies by chemical pollutants—posing a serious threat to natural resources and ecosystems worldwide [1,2,3,4]. Among these pollutants, synthetic dyes have emerged as a major concern due to their potential to pollute both surface and groundwater. The discharge of dye-laden wastewater from industrial processes has become a widespread environmental issue. Each year, the world produces an estimated 700,000 to 1,000,000 tons of synthetic dyes, of which roughly 10–15% (about 100,000 tons) are released into the environment through industrial wastewater. Most of these dyes, particularly azo and reactive dyes, are persistent, non-biodegradable, and toxic, posing serious risks to aquatic ecosystems and human health. Notable pollution incidents include the Citarum River in Indonesia, which became one of the world’s most polluted waterways; the Noyyal River in Tirupur, India, where dye discharges rendered farmland infertile; the Pearl River Delta in China, which suffered severe aquatic toxicity from benzidine-based dyes; and the Buriganga River in Bangladesh, which was contaminated by untreated dye and heavy metal effluents. Such cases underscore the urgent global need for improved wastewater treatment, stricter environmental policies, and greener alternatives in the dyeing industry to prevent ecological and public health harm [5,6,7]. With over 10,000 different dye types used across various industries for coloration, the need for advanced and efficient water treatment technologies has never been more urgent. As such, the ongoing pursuit of innovative and effective solutions for environmental remediation remains a critical global priority [8,9].

To meet the growing demands of an increasing global population, dye-intensive industries such as textiles, food, leather, paper, cosmetics, pulp and paper mills, plastics, printing, and carpet manufacturing are expanding rapidly. However, this industrial growth has led to the widespread presence of dye pollutants in wastewater effluents, which are often illegally discharged into surface water bodies, posing a serious environmental and public health risk. Synthetic dyes pose serious threats to plants, animals, and humans because of their toxic, persistent, and bio-accumulative nature. In plants, these dyes block sunlight penetration in water and interfere with chlorophyll formation, reducing photosynthesis, nutrient uptake, and overall growth. In animals, particularly aquatic species, dyes (especially azo and anthraquinone types) can accumulate in tissues and cause cellular damage, enzyme inhibition, reproductive failure, and even death. Fish and amphibians are especially vulnerable due to gill and organ toxicity. For humans, exposure through contaminated water, food, or direct contact can lead to skin allergies, respiratory irritation, liver and kidney damage, and in severe cases, cancer, since many dyes break down into carcinogenic aromatic amines. Long-term exposure can also lead to mutagenic and teratogenic effects, posing severe risks to public health and ecosystems alike [10,11,12,13,14]. The textile industry, in particular, is a major contributor—losing approximately 12% of dyes during manufacturing and dyeing processes, with nearly 20% of this loss entering water systems as waste. These colorants, often composed of complex aromatic structures and azo groups, are especially concerning due to their tendency to degrade into toxic aromatic amines, further exacerbating their environmental impact.

Textile effluents are frequently discharged into water bodies without undergoing adequate treatment, leading to significant environmental concerns. A primary issue within these effluents is the presence of synthetic dyes, which exhibit high solubility in water, even at low concentrations. This property allows them to spread easily through aquatic environments, disrupting ecological balance. The presence of these dyes impairs light penetration in water, which adversely affects photosynthetic processes and, in turn, diminishes oxygen levels—ultimately threatening the survival and biological functions of aquatic organisms. Moreover, these pollutants are toxic, carcinogenic, mutagenic, and non-biodegradable, making them particularly harmful to both ecosystems and human health [15,16,17]. The inadequate treatment of dye-laden wastewater, a result of anthropogenic industrial activities, has intensified the current water pollution crisis. Among various water pollutants, synthetic dyes are considered among the most persistent and hazardous due to their complex aromatic structures and resistance to degradation. These dyes, widely used in industries such as textiles and leather, remain in the environment for extended periods, compounding their ecological and health-related impacts.

The treatment of dye-contaminated wastewater presents a significant challenge due to the complex aromatic structures of synthetic dyes, which render them highly resistant to microbial degradation, oxidative agents, heat, water, and light. As a result, untreated dye effluents contribute to persistent environmental pollution, degrading the aesthetic quality of water bodies and posing substantial toxicity risks to aquatic organisms. These impacts can disrupt physiological processes such as growth, reproduction, and survival, ultimately affecting biodiversity and ecosystem stability. Therefore, accurately identifying the types and chemical characteristics of dye pollutants is essential for developing effective remediation strategies. Among various treatment methods, adsorption has emerged as a promising and adaptable technique for the removal of industrial dyes, offering numerous advantages in terms of efficiency, cost-effectiveness, and ease of operation in wastewater treatment applications [18,19,20,21].

Considering the aforementioned challenges, a range of physical, chemical, and biological methods—such as coagulation, reverse osmosis, photodegradation, electrochemical oxidation, ozonation, biosorption, and adsorption—have been adopted to enhance water quality by eliminating dye pollutants. The removal of dye molecules from aquatic systems is of critical importance due to their toxicity, persistence, and adverse ecological impacts. Each method has been explored to mitigate the environmental hazards posed by dye-laden effluents. Among these, adsorption stands out as one of the most widely applied and effective techniques for dye removal. Adsorption is a well-known separation technique in which specific chemical substances, known as adsorbates, accumulate on the surface of a solid material called the adsorbent. Its popularity stems from its operational simplicity, cost-effectiveness, broad applicability, and minimal generation of hazardous by-products. Moreover, adsorption does not require sophisticated equipment or highly trained personnel, making it a practical solution for wastewater treatment across various industrial sectors. It is particularly effective for the removal of both organic and inorganic hazardous contaminants from polluted water. The adsorption method offers several key advantages over other pollutant treatment approaches such as chemical oxidation, coagulation, membrane filtration, and biological degradation. It is widely preferred for removing dyes, heavy metals, and organic contaminants because of its high efficiency, operational simplicity, and versatility. Unlike chemical or biological treatments, adsorption does not produce harmful byproducts or require complex pretreatment. It effectively removes both biodegradable and non-biodegradable pollutants, even at low concentrations, where other methods often fail. Additionally, the process operates under mild conditions (ambient temperature and pressure) and allows for the regeneration and reuse of adsorbents like activated carbon, biochar, and nanomaterials, making it cost-effective and environmentally friendly. Compared to membrane filtration, adsorption has lower energy demands and less fouling, and unlike oxidation processes, it avoids secondary pollution from residual chemicals. These features make adsorption a clean, efficient, and sustainable choice for wastewater and industrial effluent treatment [22,23,24,25].

A wide variety of materials have been employed for the adsorption of dyes from aqueous solutions. These include metallic, bio-based, organic, and inorganic adsorbents, each explored for their potential in effectively removing dye pollutants. Notable examples include biomass-derived carbons [26,27], nanometal oxides [28,29], clays [30,31], and cellulose or chitosan-based composites [32,33]. Despite promising results, many conventional adsorbents face limitations related to cost, adsorption efficiency, and selectivity toward specific dye molecules. This highlights the need for continued research and development of novel adsorbent materials tailored for wastewater treatment applications. The selection of an appropriate adsorbent is a critical factor, encompassing both naturally occurring substances—such as zeolites, clays, activated carbons, and metal oxides—and next-generation materials including nanoparticles, hydrogels, aerogels, biosorbents, and polymeric nanocomposites. These emerging materials offer promising alternatives due to their enhanced surface area, tunable properties, and potential for high adsorption capacities.

The use of activated carbons (ACs) in adsorption technologies has a long-established history, stemming from both natural and synthetic precursors. As an adsorbent, activated carbon is recognized for its high efficiency and environmental compatibility. It has been widely applied in the removal of organic and inorganic pollutants from industrial wastewater—including effluents from pharmaceutical, textile, paper, and printing industries—through adsorption mechanisms. ACs are favored due to their exceptionally large surface area, high porosity, significant pore volume, diverse surface functional groups, and overall ease of handling and availability. These properties contribute to their high adsorption capacity and adaptability across various treatment systems. Owing to their tunable surface chemistry and modifiability, activated carbons are considered one of the most effective adsorbents. Structurally, AC consists of processed carbon with a network of micropores and mesopores, which significantly enhance surface area and facilitate adsorption and chemical interactions. However, the high production cost associated with conventional ACs has driven the search for alternative, cost-effective precursors—particularly from renewable biomass sources. Bio-sorbents derived from agricultural waste and other plant-based materials offer a promising solution [34,35,36]. These materials are typically abundant, inexpensive, and often available as by-products of other industrial processes. Numerous studies have demonstrated the potential of plant-derived bio-sorbents to effectively remove toxic contaminants from water and wastewater, offering a sustainable alternative to conventional adsorbents.

A wide range of biomass-derived raw materials—such as paper sludge, corn straw, tea leaves, rice husks, municipal waste, seed shells, palm leaves, bamboo, wheat husks, animal manure, sawdust, fruit peels, and cotton stalks—have been frequently explored as feedstocks for the production of low-cost adsorbents [37,38]. To assess the efficiency of these materials in dye removal, various experimental parameters have been systematically investigated. Key variables such as pH, adsorbent dosage, initial dye concentration, contact time, and temperature are commonly evaluated to study adsorption equilibrium, kinetics, and thermodynamic behavior. The synthesized adsorbent membranes are typically characterized using advanced analytical techniques. Scanning electron microscopy (SEM) is used to analyze surface morphology; fourier transform infrared (FTIR) spectroscopy identifies functional groups; thermogravimetric analysis (TGA) determines thermal stability; and Brunauer–Emmett–Teller (BET) analysis provides surface area and porosity data—together offering comprehensive insights into the structural and functional properties of the developed adsorbents. The main objective of this review is to provide a comprehensive overview of the development and application of bio-derived carbons and raw biomass powders as efficient adsorbents for dye removal from wastewater.

Articles were selected based on the following criteria: (i) use of raw biomass, biochar, or biomass-derived activated carbon as adsorbents; (ii) application toward synthetic dye removal from aqueous media; (iii) availability of quantitative adsorption data (adsorption capacity, kinetics, isotherms, or regeneration performance); and (iv) relevance to sustainability, cost-effectiveness, and environmental applicability. Studies focusing solely on non-adsorptive dye degradation techniques were excluded. The main focus of this manuscript is to critically review the development, characterization, adsorption behavior, and performance of bio-derived carbons and raw biomass materials for dye removal, highlighting adsorption mechanisms, influencing parameters, regeneration ability, and future prospects for large-scale wastewater treatment applications.

## 2. Classification of Dyes

**Figure 1:**
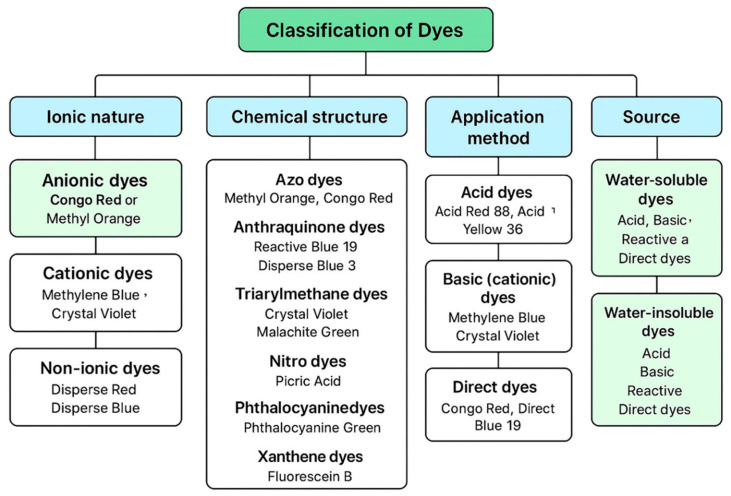
Classification of dyes.

Dyes, as stated earlier, can be obtained from natural sources such as vegetable matter, mineral or insects or are manufactured in the factory from petrochemical feedstock. It may, however, be recalled that the first synthetic dye (Mauveine) by Perkin was made from Coal tar. Amongst natural dyes, indigo is well known for its brilliant blue color and was obtained by fermenting the leaves of a plant. The red colored lac dye is extracted from lac, a resinous protective secretion of a tiny insect. Dyes can be classified based on several criteria, including their ionic nature, chemical structure, application method, solubility, and source (Figure 1).

Based on ionic nature, dyes are categorized into cationic, anionic, and non-ionic dyes. Cationic dyes (e.g., methylene blue, crystal violet, malachite green) carry positive charges in aqueous solutions and exhibit strong electrostatic attraction toward negatively charged adsorbent surfaces. Anionic dyes (e.g., Congo red, methyl orange, reactive and acid dyes) possess sulfonate or carboxylate groups and preferentially interact with protonated or positively charged adsorbents. Non-ionic dyes lack formal charge and are typically removed through hydrogen bonding, π–π interactions, or hydrophobic forces. According to chemical structure, dyes are classified into azo, anthraquinone, triarylmethane, indigoid, xanthene, and phthalocyanine dyes. Among these, azo dyes, characterized by one or more –N=N– bonds, represent the largest dye class used industrially due to their vivid colors, low cost, and chemical stability. However, their degradation often produces toxic aromatic amines, making their removal environmentally critical. Based on application and fiber affinity, dyes are grouped into acid, basic, direct, reactive, disperse, vat, sulfur, and azoic dyes. Reactive dyes form covalent bonds with cellulose fibers, making them highly water-soluble and difficult to remove, whereas disperse dyes are sparingly soluble and mainly used for synthetic fibers. Vat and sulfur dyes are insoluble in water and require chemical reduction during application. Dyes may also be classified by origin as natural dyes (derived from plants, insects, or minerals) or synthetic dyes (petroleum-based). Synthetic dyes dominate modern industries due to their color consistency, stability, and scalability but pose significant environmental risks due to poor biodegradability. Understanding these classifications is essential for designing efficient adsorption systems and tailoring bio-derived adsorbents for selective dye removal [26,27,28,29,30,31,32,33].

The efficiency of dye adsorption onto bio-derived adsorbents is governed by several interrelated physicochemical parameters. Initial dye concentration plays a critical role by providing the driving force required to overcome mass transfer resistance; higher concentrations generally increase adsorption capacity until saturation of active sites occurs. Solution pH significantly influences adsorption by altering the surface charge of the adsorbent and the ionization state of dye molecules, thereby affecting electrostatic interactions. Temperature impacts adsorption thermodynamics and kinetics, where endothermic processes benefit from elevated temperatures due to enhanced molecular mobility, while exothermic adsorption favors lower temperatures. The adsorbent dosage determines the availability of active sites, with increasing dosage improving removal efficiency but potentially reducing adsorption capacity per unit mass due to site underutilization. Additionally, the presence of surface functional groups (e.g., hydroxyl, carboxyl, amino groups) governs adsorption mechanisms such as hydrogen bonding, electrostatic attraction, π–π interactions, and ion exchange. Contact time influences adsorption equilibrium, with rapid uptake typically occurring during the initial stages due to abundant vacant sites, followed by slower diffusion-controlled processes. Optimization of these parameters is essential for maximizing dye removal efficiency and practical wastewater treatment performance [25,26,27,28,29,30].

## 3. Investigation of Adsorption Behavior of Raw and Carbonized Materials for Dye Removal

**Figure 2:**
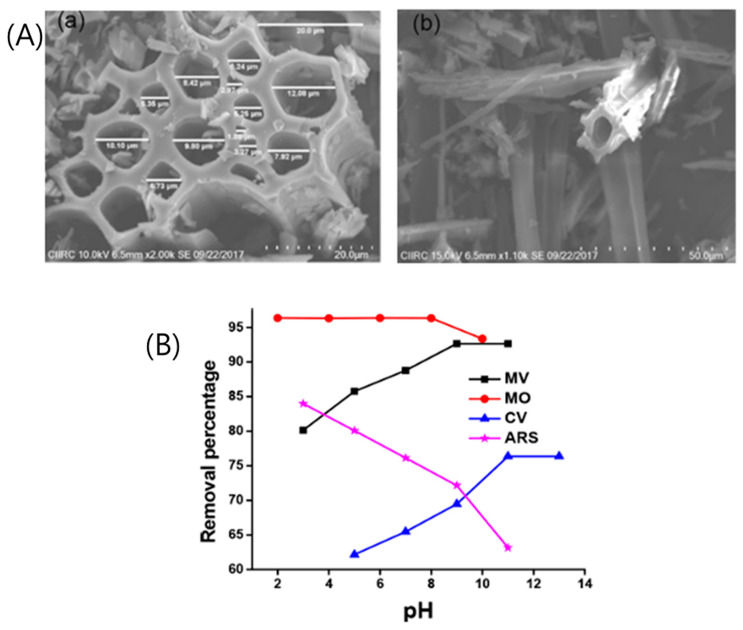
(**A**) SEM images of (**a**) BFC 20 μm, (**b**) BFC at 50 μm; and (**B**) Variation in removal efficiency of BFC with pH of different dyes utilized in the study. Reproduced with permission from [4].

S. Olivera et al. [4] developed hollow, tube-like banana fiberr carbon (BFC) materials for the adsorptive removal of both cationic and anionic dyes from water, including methyl violet (MV), crystal violet (CV), methyl orange (MO), and alizarin red S (ARS). Raw banana fibers were calcined under a nitrogen atmosphere at 550 °C for one hour, producing banana fibre carbon (BFC). As illustrated in Figure 2A, the resulting BFC displayed a micrometer-sized, tubular morphology consistent with previous reports [39,40], with pore diameters ranging from 1.88 to 12.08 μm. This open-ended, porous structure facilitates the effective diffusion and adsorption of dye molecules. Despite its relatively low specific surface area (8.09 m^2^/g), the BFC contained numerous surface functional groups that enhanced dye uptake through favorable interactions at optimal pH conditions. The material demonstrated notable adsorption capacities of 85.65, 78.95, 65.78, and 65.07 mg/g for MV, MO, CV, and ARS, respectively. Regeneration studies, performed by treating dye-loaded BFC with 0.01 N HCl, revealed only a modest reduction in removal efficiency after three adsorption–desorption cycles—decreasing from 89.29, 76.36, 92.81, and 78.33% to 81.28, 69.75, 85.25, and 70.48% for MV, CV, MO, and ARS, respectively (Figure 2B). The ability to use carbon derived directly from banana fibers without additional activation highlights the material’s potential as an energy-efficient and cost-effective adsorbent for wastewater treatment.

**Figure 3:**
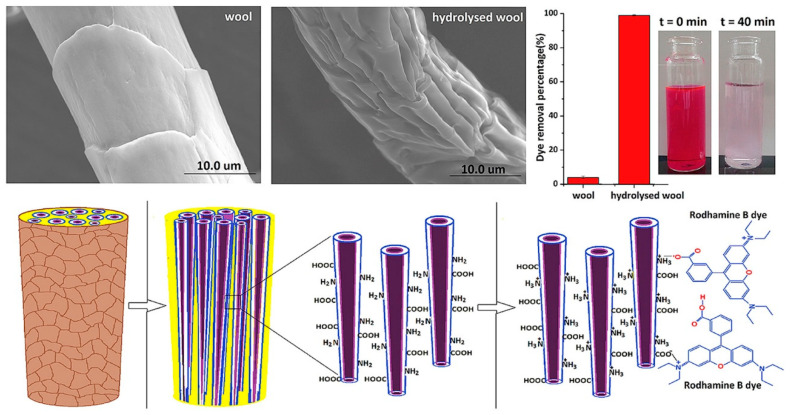
Surface hydrolysed keratin protein fibers. Reproduced with permission from [9].

Tissera et al. [9] developed innovative surface-hydrolyzed keratin protein fibers derived from merino wool, offering an efficient bio-based material for the removal of Rhodamine B dye from aqueous solutions (Figure 3). The surface hydrolysis treatment disrupted the protective cuticle cells that typically envelop the hierarchical microstructure of wool fibers, thereby enhancing their adsorption performance. This process exposed the ortho-cortex region of the fibers, significantly increasing the availability of free amine and other reactive surface functional groups. Among the different alkali-treated samples, fibers hydrolyzed with 0.5 M NaOH demonstrated superior performance, achieving nearly 95% Rhodamine B removal and exhibiting a maximum adsorption capacity of 294 mg/g at 298 K in the presence of 3.5% acetic acid—representing one of the highest reported capacities under comparable conditions. The adsorption mechanism was primarily governed by chemical interactions between the dye molecules and the exposed amine and carboxylic groups, as well as by charge-driven adsorption–desorption processes influenced by the protonation and deprotonation of amine sites. A notable advantage of these keratin fibers is their excellent reusability over multiple adsorption cycles. SEM imaging revealed that untreated wool retained its characteristic tubular structure with distinct scale-like cuticle layers, while progressively stronger alkaline treatment (0.5 M NaOH) transformed the surface into a fibrillar morphology at the submicrometer scale, diminishing the visibility of cuticle cells—an observation further confirmed through AFM analysis [41].

**Figure 4:**
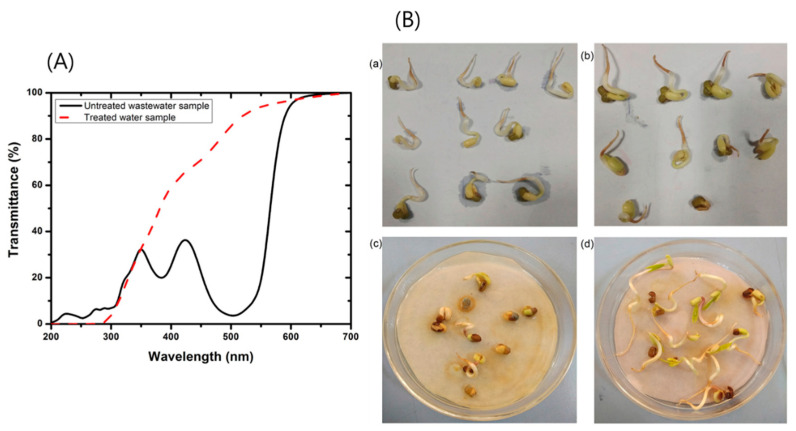
(**A**) Change in UV-visible transmittance spectra of untreated and treated real textile wastewater; and (**B**) Germinated green gram seed in treated water samples: (**a**) treated mixed RR21 and RO16 dye contaminated water sample, (**b**) treated mixed RR21, RO16 and RB19 dye contaminated dye water sample, (**c**) untreated textile wastewater, and (**d**) treated textile wastewater. Reproduced with permission from [42].

Mishra et al. [42] explored the bacterial decolorization of azo dyes (Reactive Red-21, Reactive Orange-16) and an anthraquinone dye (Reactive Blue-19) using *Pseudomonas aeruginosa* SVM16—identified through nucleotide sequencing—in combination with jackfruit seed (JFS) powder as a co-substrate [43,44,45]. Ripened jackfruits were procured from a local vegetable market in Saharanpur, India, and their seeds were manually separated, washed with double-distilled water (DDW), and oven-dried at 50 °C. The dried seeds were ground into powder, and 1 g of this powder was dissolved in 10 mL of sterilized DDW for use in the experiments. The bio-decolorization process achieved a tenfold reduction in residual carbon content in treated water compared to systems using yeast extract as a nutrient source. Optimization via response surface methodology (RSM) revealed that *P. aeruginosa* SVM16 exhibited excellent salt tolerance and maximum decolorization efficiency across a pH range of 6–9. When applied to actual textile wastewater, the method achieved a 77.32 ± 0.5% reduction in color intensity, corresponding to an initial ADMI (American Dye Manufacturers Institute) value decrease from 467.95 ± 8 (Figure 4A) [46,47]. Toxicity assays confirmed that the treated effluent was non-toxic, supporting 100% germination of *Vigna radiata* seeds (Figure 4B). Overall, the findings highlight the potential of JFS powder as a low-cost, eco-friendly co-substrate for the bioremediation of dye-contaminated textile wastewater.

**Figure 5:**
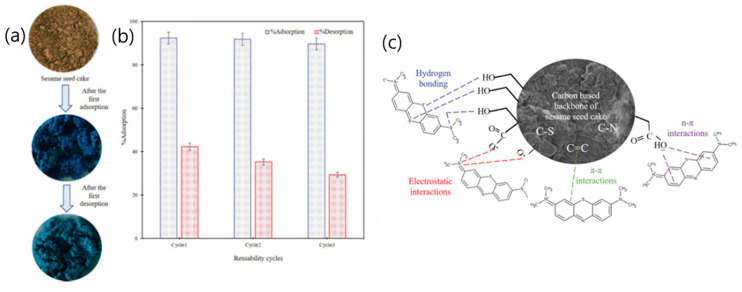
(**a**) Physical change; (**b**) reusability cycles of sesame seed cake; and (**c**) proposed interaction mechanism between methylene blue and sesame seed cake. Reproduced with permission from [37].

Yilmaz et al. [37] investigated the adsorption of methylene blue dye using a novel, low-cost bio-sorbent derived from raw sesame seed cake. The sesame seed cake was first dried at 50 °C for 24 h to eliminate moisture and then ground to an average particle size of approximately 100 mesh. Adsorption experiments were conducted for 25 min using an initial methylene blue concentration of 200 mg/L, while varying key parameters such as temperature (20–40 °C), pH (3–9), and adsorbent dosage (1–3 g/100 mL). SEM images revealed that the raw sesame seed cake exhibited an amorphous texture with irregular cubic morphology and heterogeneous pores, which enhanced dye adsorption efficiency [48,49,50]. Optimization of the adsorption process was performed using response surface methodology (RSM) and the Box–Behnken design (BBD), considering pH, adsorbent dosage, and temperature as independent variables. Statistical evaluation through ANOVA confirmed the model’s reliability and the significant influence of these parameters on dye removal efficiency. The results indicated that adsorption of methylene blue onto sesame seed cake was far more dominant than desorption (Figure 5a,b), primarily due to strong hydrogen bonding between dye molecules and the functional groups on the cake surface (Figure 5c). The adsorption rate constant (α = 26.6273 mg/g·min) was markedly higher than the desorption rate constant (β = 2.8425 g/mg), corroborating this observation [51]. Reusability tests demonstrated that sesame seed cake maintained substantial adsorption capacity across three consecutive cycles (with the influence order: pH > adsorbent dosage > temperature), although desorption efficiency remained limited due to the inherent nature of the adsorption mechanism.

Nisreen S. Ali et al. [52] investigated the use of raw date seeds—an abundant and low-cost agricultural byproduct—as an eco-friendly bio-adsorbent for the removal of methyl violet dye from wastewater, thereby addressing a major environmental challenge. The date seeds were ground using a steel mill to a particle size of less than 300 μm, followed by drying at 100 °C for 24 h to eliminate residual moisture before use in adsorption experiments. Adsorption equilibrium was achieved within 1 h under ambient pH conditions, and the experimental data were found to fit the Langmuir isotherm model, yielding a maximum biosorption capacity (q_max_) of 59.5 mg/g [53]. Kinetic analysis revealed that the sorption process followed a pseudo–second-order model, with a rate constant of 7.61 g/mg·min, suggesting chemisorption as the dominant mechanism. Thermodynamic evaluation further confirmed that the adsorption process was both endothermic and spontaneous. Overall, the findings demonstrate that untreated date seed powder serves as an efficient, inexpensive, and sustainable bio-sorbent for methyl violet dye removal, offering a practical solution for wastewater decolorization and reuse.

Salman et al. [5] examined the potential of Cycas leaf powder (CL), an inexpensive agro-waste material, as a natural adsorbent for the removal of malachite green (MG) and indigo carmine (IC) dyes from contaminated water. The collected leaves were thoroughly washed, sun-dried for 48 h, and subsequently oven-dried at 95 °C for another 48 h. The dried material was then crushed, ground, and sieved to obtain a fine powder with a particle size of approximately 100 μm. Surface analysis revealed that the CL powder possessed a smooth and relatively homogeneous texture with minor porosity. Elemental composition analysis indicated notable changes after dye adsorption: for MG-loaded samples, 72.3% carbon, 13.41% oxygen, and 12.09% scandium were detected, while IC-loaded samples contained 71.79% carbon, 15.86% oxygen, and 11.74% scandium—confirming successful adsorption of the organic dyes. Adsorption data best fitted the Freundlich isotherm model, suggesting multilayer adsorption on heterogeneous surfaces, while kinetic studies showed good agreement with the pseudo-first-order model [54,55]. Thermodynamic analysis revealed that the adsorption of MG was exothermic and non-spontaneous, whereas that of IC was endothermic. Moreover, adsorption efficiency increased with higher initial dye concentrations. These findings collectively demonstrated that Cycas leaf powder is an effective, low-cost bio-sorbent with promising potential for the remediation of dye-contaminated wastewater.

Laggoun et al. [56] explored the use of cockle shell (CS) powder as a natural, low-cost bio-sorbent for the removal of toxic textile dyes—Terasil Red (TR) and Cibacron Green H3G (CG-H3G)—from industrial effluents in Algeria. Owing to their abundance on Algerian beaches, cockle shells represent a sustainable and readily available adsorbent source. The preparation of the CS adsorbent involved several steps: shells were repeatedly washed with distilled water to remove impurities, air-dried for 24 h, and subsequently crushed using a mechanical grinder. The resulting material was sieved to obtain a fine powder with a particle size of ≤125 µm, which was then utilized in both single and binary dye biosorption experiments [57]. Morphological characterization using SEM and EDX revealed a non-uniform, irregular particle structure with varied porosity. After dye adsorption, SEM images showed that TR and CG-H3G molecules adhered effectively to the CS surface. EDX spectra confirmed changes in elemental composition post-adsorption: the calcium carbonate content varied, strontium disappeared, and new elements appeared—phosphorus (0.27%) and chlorine (0.36%) in TR-loaded samples; sodium (0.27%) and sulfur (0.18%) in CG-H3G-loaded samples—verifying successful dye uptake. Adsorption behavior followed the Langmuir isotherm model and pseudo–second-order kinetics, indicating monolayer chemisorption. Thermodynamic analysis showed that the process was spontaneous and exothermic within the temperature range of 22–31 °C. Furthermore, CG-H3G exhibited higher adsorption selectivity compared to TR in both single and binary dye systems, with equilibrium achieved within 6–45 min. Overall, cockle shell powder proved to be an efficient, eco-friendly bio-sorbent for textile wastewater treatment.

**Figure 6:**
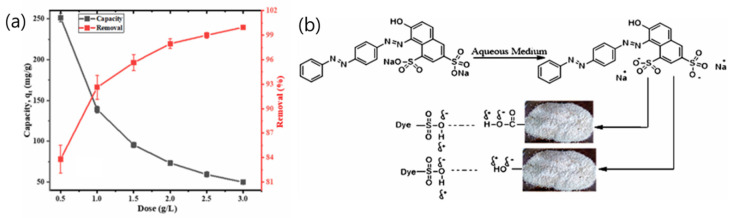
(**a**) PP dosage on adsorption capacity; and (**b**) Plausible mechanisms of AR73 dye adsorption on PP. Reproduced with permission from [58].

Zohra et al. [58] investigated the potential of potato peel powder (PP)—an abundant agricultural waste—as a sustainable and low-cost adsorbent for the removal of Acid Red 73 (AR73), a commonly used leather dye, from tannery wastewater. Given that acid dyes are frequently employed in leather processing, this study aimed to address a significant source of industrial pollution through eco-friendly bio-sorption. Fresh potato peels were oven-dried at 60 °C for 48 h, ground using a stainless-steel grinder, and stored in airtight containers for subsequent use. The powdered peels were then employed to remove AR73 from both synthetic aqueous dye solutions and actual tannery effluents. SEM micrographs revealed a rough, porous morphology that favors dye adsorption, while XRD analysis exhibited a broad hump in the 2θ = 15–25° range and a weak peak at 2θ = 17.16°, confirming the amorphous carbonaceous structure of the material [59,60]. The maximum adsorption capacity of PP for AR73 was 258.39 mg/g, with adsorption equilibrium achieved within 30 min. Isotherm modeling showed that the data fit both the Langmuir (R^2^ = 0.989) and Freundlich (R^2^ = 0.993) models, though the Freundlich model provided a better correlation, indicating heterogeneous surface adsorption. Kinetic analysis revealed excellent conformity with the pseudo–second-order model (R^2^ = 0.999), suggesting that chemisorption governed the dye uptake mechanism. Thermodynamic parameters confirmed that the adsorption process was spontaneous, exothermic, and feasible. In tests with real tannery wastewater, PP achieved approximately 98.17 ± 0.58% dye removal with an adsorption capacity of 137.39 ± 2.46 mg/g, while also significantly reducing TDS, EC, BOD_5_, and COD to acceptable discharge levels. However, regeneration experiments revealed a gradual decline in performance, with capacity decreasing from 86.73 ± 2.61 mg/g to 37.49 ± 2.83 mg/g after three reuse cycles (Figure 6a,b). Overall, potato peel powder demonstrated excellent adsorption efficiency and environmental compatibility, making it a biodegradable and cost-effective adsorbent for dye-laden industrial effluents, though improvements in reusability are still needed.

## 4. Sustainable Dye Removal Using Activated Carbon Adsorbents

**Figure 7:**
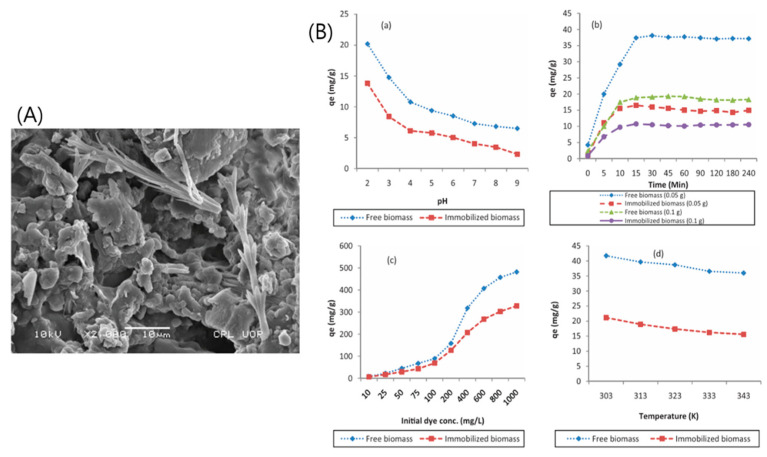
(**A**) SEM analysis of *Trogoderma granarium* de-oiled biomass; and (**B**) Effect of (**a**) pH, (**b**) contact time and biomass dosage, (**c**) initial dye concentration and (**d**) temperature on the removal of Drimarine Yellow HF-3GL dye by using *Trogoderma granarium* de-oiled biomass. Reproduced with permission from [1].

Iram Naz et al. [1] investigated the de-oiled biomass of *Trogoderma granarium* (khapra beetle larvae) as a low-cost, novel bio-sorbent for the removal of Drimarine Yellow HF-3GL dye from water. This marks the first reported use of this insect for both biofuel production and wastewater treatment. The larvae, with a high fat content of 53%, underwent Soxhlet extraction with n-hexane at 60 °C, completing fat removal in 90 min—indicating strong potential as a biofuel feedstock [61,62]. The residual biomass was tested in free and immobilized forms for dye adsorption. Optimal performance was observed at pH 2 and 30 °C, with a maximum adsorption capacity of 481.9 mg/g and equilibrium reached within 15 min. Adsorption decreased at higher pH due to electrostatic repulsion by OH^−^ ions, aligning with known behavior of anionic dye systems [63]. Pretreatment with acids or alkali offered no significant improvement, suggesting that fat extraction alone enhances surface properties. SEM analysis revealed a porous and heterogeneous structure (Figure 7A), contributing to efficient dye uptake. Desorption studies showed that 0.4% NaOH achieved 78.7% dye release, indicating good reusability (Figure 7B). Overall, the *T. granarium* biomass outperformed many lignocellulosic adsorbents [64,65], making it a promising candidate for sustainable dye removal in wastewater treatment.

Mahmoodi et al. [34] synthesized low-cost mesoporous activated carbons (ACs) from agricultural bio-wastes—kiwi peel (KP), cucumber peel (CP), and potato peel (PP)—designated as AC_KP, AC_CP, and AC_PP, respectively. These bio-derived materials were developed as sustainable adsorbents for dye removal from aqueous solutions. To enhance adsorption performance, the prepared carbons underwent KOH surface activation by mixing the adsorbents with a saturated KOH solution (121 g KOH in 100 g water) at a 1:5 mass-to-volume ratio for 5 h at 70 °C. After activation, the materials were washed with diluted HCl, rinsed with double-distilled water until neutral pH (≈7), and oven-dried at 60 °C. FESEM revealed that the raw peels exhibited dense, non-porous surfaces with irregularly distributed granular particles, whereas activation generated a well-developed porous structure with diverse pore sizes and morphologies [66,67,68]. The zeta potential values (−48.7, −43.5, and −41.8 mV for AC_KP, AC_CP, and AC_PP, respectively) indicated highly negative surface charges, enhancing electrostatic interactions with cationic dyes. Kinetic studies were conducted using 0.025 g of adsorbent in 50 mL of methylene blue (MB) solution at natural pH (6.3). Increasing the adsorbent dosage from 0.005 g to 0.025 g resulted in higher adsorption capacity (qₑ), attributed to the greater number of available binding sites. The adsorption process was identified as multi-stage, involving external mass transfer followed by intra-particle diffusion within the porous structure. Thermodynamic analysis showed that MB adsorption onto all ACs was endothermic, spontaneous, and dominated by physical sorption. The materials effectively removed dyes not only from single systems but also from binary (MB + malachite green, MB + rhodamine B) and ternary (MB + MG + RhB) mixtures. The Langmuir isotherm and pseudo–second-order (PSO) kinetic model best described the adsorption behavior, indicating monolayer chemisorption. Furthermore, the Artificial Neural Network (ANN) model accurately predicted adsorption capacities with a correlation coefficient (R^2^) of 0.996, demonstrating excellent agreement between experimental and simulated results. Overall, this study highlights the effectiveness of KOH-activated carbons derived from fruit and vegetable wastes as efficient, eco-friendly adsorbents for complex dye-contaminated wastewater treatment.

Masoudian et al. [11] reported the ultrasonic-assisted simultaneous removal of Congo Red (CR) and Phenol Red (PhR) dyes from aqueous solutions using titanium dioxide (TiO_2_) nanoparticles (NPs) supported on activated carbon derived from watermelon rind (WR)—a sustainable bio-waste material. The composite adsorbent, denoted as TiO_2_–NPs–ACWR, was synthesized to combine the photocatalytic efficiency of TiO_2_ with the high surface area and porosity of carbonaceous substrates. SEM analysis revealed that TiO_2_ nanoparticles were uniformly dispersed across the highly porous activated carbon framework, providing a large number of active sites for dye adsorption. The composite exhibited an impressive specific surface area of 667.82 m^2^/g, thereby, confirming its suitability as an efficient adsorbent. Adsorption data were analyzed using several isotherm models, including Langmuir, Freundlich, and Temkin [69,70]. Among these, the Langmuir model best described the experimental results, indicating monolayer adsorption on a homogeneous surface with finite adsorption sites of uniform energy distribution. The study demonstrated that dye removal using a relatively small amount of TiO_2_–NPs–ACWR occurred rapidly and efficiently, achieving high sorption capacities for both CR and PhR under ultrasonic irradiation. The synergistic effect between TiO_2_ nanoparticles and the activated carbon matrix enhanced adsorption kinetics and capacity, making TiO_2_–NPs–ACWR a promising and sustainable material for wastewater purification and dye removal applications.

Bazan-Woźniak et al. [6] developed activated bio-carbons from the residues of supercritical extractions of four plant materials—raspberries, blackcurrant, nettle, and green tea. These bio-carbons were evaluated as sorbents for removing organic pollutants (methylene blue and methyl red) from aqueous solutions and nitrogen dioxide (NO_2_) from air. The study also examined how the choice of precursor influenced the physicochemical and sorption properties of the resulting activated bio-carbons. The preparation process involved impregnating the precursors with a sodium carbonate (Na_2_CO_3_) solution at an activator-to-precursor weight ratio of 2:1, drying them to constant mass at 125 °C, and subsequently thermally treating them in a nitrogen atmosphere (flow rate: 0.300 L/min). Among the materials produced, the raspberry-derived activated carbon (RAC) exhibited the highest surface area (380 m^2^/g), while those obtained from blackcurrant, nettle, and green tea ranged between 250 and 352 m^2^/g. All samples displayed comparable acid–base surface properties. Sorption tests revealed that each bio-carbon adsorbed methylene blue more effectively than methyl red. This behavior was attributed to the larger molecular mass and stronger surface affinity of methylene blue, leading to faster adsorption on the external surface rather than within the pores of the adsorbent. The adsorption data were analyzed using the linearized Langmuir and Freundlich isotherm models, with the Langmuir equation based on the assumption of a uniform distribution of energetically equivalent adsorption sites, each capable of binding one adsorbate molecule [71]. The raspberry-based activated bio-carbon demonstrated the highest adsorption capacities, ranging from 85 to 146 mg/g for methylene blue and 70 to 103 mg/g for methyl red. For gaseous adsorption, NO_2_ uptake increased notably under humid conditions (70% relative humidity), with sorption capacities varying from 25 to 81 mg. Overall, the study concluded that activated bio-carbons derived from plant extraction residues—especially from raspberry waste—represent efficient, low-cost adsorbents for both liquid-phase (methylene blue and methyl red) and gas-phase (NO_2_) pollutants. Despite their moderate surface areas, these materials exhibited strong adsorption efficiencies consistent with the Langmuir and Freundlich models, and their adsorption kinetics were best described by the pseudo-second-order model.

**Figure 8:**
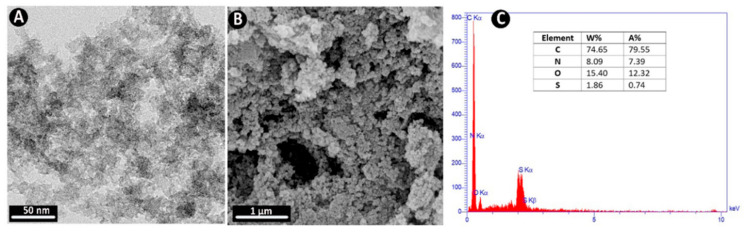
(**A**) HR-TEM; (**B**) FE-SEM; and (**C**) EDS of prepared activated carbon. Reproduced with permission from [10].

Sardi et al. [10] conducted a study to assess the potential of activated carbon (AC) derived from dried palm wood for the removal of cationic dyes—Basic Blue 41 and Basic Red 46—from aqueous solutions. The preparation involved mixing palm wood powder with phosphoric acid (H_3_PO_4_) at a 1:3 weight ratio (carbon powder:H_3_PO_4_) and leaving the mixture at room temperature for 24 h. Subsequently, the mixture was carbonized in an electric furnace under an air atmosphere at 600 °C for 3 h. Zeta potential measurements indicated a negatively charged surface (−27.7 mV) for the activated carbon, favoring electrostatic attraction between the negatively charged adsorbent surface and the positively charged dye molecules. HR-TEM confirmed that the resulting porous carbon possessed an amorphous and relatively uniform pore structure [72,73]. SEM further revealed a highly porous morphology with a large surface area (Figure 8), making the material particularly suitable for adsorption applications. Nitrogen adsorption–desorption analysis at 77 K characterized the porosity of the activated carbon, yielding a total pore volume of 1.74 cm^3^ g^−1^ (at p/p_0_ = 0.990), a BET surface area of 880.29 m^2^ g^−1^, and an average pore diameter of 7.9 nm. This high surface area contributed significantly to the material’s strong adsorption performance. The adsorption of both Basic Blue 41 and Basic Red 46 followed the pseudo-second-order kinetic model, indicating that the rate-limiting step was chemisorption involving valence forces through sharing or exchange of electrons between adsorbent and adsorbate [74]. The process was further validated using real water samples collected from the Karoon River and local tap water. These samples were filtered to remove suspended solids and then spiked with different concentrations of the target dyes. Under optimized conditions, the activated carbon demonstrated high removal efficiency, confirming its practicality for real-world water treatment applications. When compared to other adsorbents reported in the literature, the palm wood-derived activated carbon exhibited higher adsorption capacities for both dyes. This superior performance was attributed to its large surface area, uniform pore structure, and favorable surface charge characteristics [75,76]. Overall, the study concluded that activated carbon synthesized from palm wood waste represents a highly effective, low-cost adsorbent with strong potential for removing cationic dyes from aqueous environments.

**Figure 9:**
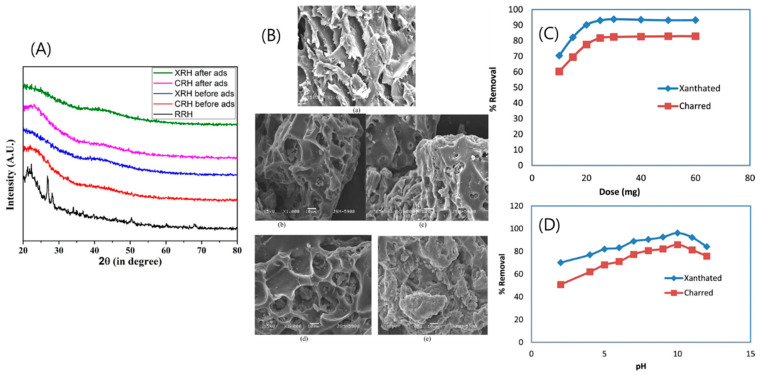
(**A**) X-ray diffraction patterns; (**B**) SEM images of (**a**) RRH, (**b**) CRH, (**c**) CRH (ads), (**d**) XRH and (**e**) XRH (ads); (**C**) Removal versus adsorbent dose; and (**D**) Effect of pH for adsorption of CV dye onto CRH and XRH. Reproduced with permission from [77].

Homagai et al. [77] investigated the use of xanthate-functionalized charred rice husk (XRH) as an efficient adsorbent for removing Crystal Violet (CV) dye from aqueous solutions. In this approach, rice husks were pre-treated with acid to eliminate lignin, hemicellulose, and crystalline components, thereby increasing porosity and surface area. Modified rice husk was identified as a promising adsorbent for wastewater treatment due to its insolubility in water, strong chemical stability, and excellent mechanical strength. The study compared the adsorption efficiency of pre-treated Charred Rice Husk (CRH) and chemically modified Xanthated Rice Husk (XRH) for CV dye removal. Pyrolysis was conducted by treating rice husk with concentrated sulfuric acid followed by heating in an inert nitrogen atmosphere, resulting in structural alterations observable by the disappearance of sharp peaks in XRD spectra (Figure 9A). Following the xanthation process, CRH underwent significant improvements in surface texture, chemical functionality, and biodegradability. FE-SEM analysis revealed that the originally honeycomb-like structure of CRH transformed into a non-uniform, rough morphology in XRH (Figure 9B), with numerous irregular pores providing increased surface area and additional active binding sites. These modifications enhanced the adsorption capacity of XRH. After dye adsorption, both CRH (ads) and XRH (ads) displayed honeycomb structures filled with dye residues, confirming successful dye uptake. The presence of flake-like deposits and a smoother surface post-adsorption indicated strong physicochemical interactions between the CV dye ions and the functional groups on the bio-sorbent surfaces. Adsorption performance was found to be pH-dependent, with maximum dye uptake observed above pH 9 for XRH and above pH 10 for CRH (Figure 9C,D). Below the point of zero charge (pHpzc), the adsorbent surfaces became positively charged, leading to electrostatic repulsion with the cationic CV dye and reduced adsorption efficiency. Conversely, at pH levels above pHpzc, the adsorbent surfaces were negatively charged, favoring electrostatic attraction and enhancing adsorption through ion-exchange mechanisms. Overall, the study demonstrated that xanthate-modified rice husk (XRH) exhibited superior adsorption capacity compared to unmodified charred rice husk (CRH), suggesting that XRH is a promising, eco-friendly, and cost-effective bio-sorbent for the removal of Crystal Violet dye from aqueous environments.

**Figure 10:**
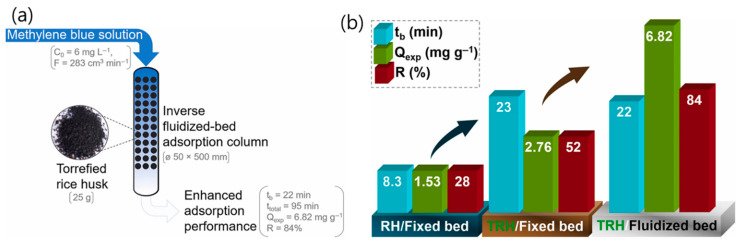
(**a**) Bio-adsorption of the dye solution is demonstrated in an inverse fluidized-bed column; and (**b**) The representative of the adsorption characteristics on the RH and TRH in the fixed-bed and inverse fluidized-bed adsorption columns. Adsorption conditions: C_0_ of 6 mgL^−1^, F of 100 cm^3^ min^−1^ (the fixed-bed adsorption column) and 283 cm^3^ min^−1^ (the inverse fluidized-bed adsorption column). Reproduced with permission from [78].

Hummadi et al. [78] introduced, for the first time, the use of an inverse fluidized-bed bio-adsorption column employing torrefied rice husk (TRH) for the removal of methylene blue (MB) dye from aqueous solutions. In their experimental procedure, approximately 10 g of dried rice husk (RH) per batch was treated with 100 mL of 15 M H_2_SO_4_ at 600 °C under continuous stirring at 200 rpm for 4 h to produce the torrefied adsorbent. The study demonstrated that using TRH in an inverse fluidized-bed adsorption system significantly enhanced the removal efficiency of methylene blue compared to a conventional fixed-bed column utilizing untreated rice husk (Figure 10a). This setup allowed for improved mass transfer and better contact between the adsorbent and dye molecules, leading to higher adsorption rates. Optimal adsorption performance in the inverse fluidized-bed column was achieved at a superficial velocity of 0.00224 m/s (equivalent to a flow rate of 283 cm^3^/min), resulting in an adsorption capacity of 6.82 mg/g and an overall dye removal efficiency of 84% (Figure 10b). These findings confirmed the technical feasibility and superior performance of the TRH-based inverse fluidized-bed system for dye removal. This study’s novelty lies in its first-time demonstration of the inverse fluidized-bed bio-adsorption process for dye remediation, as well as its comprehensive evaluation of rice husk performance across three adsorption systems—dried, torrefied, and untreated forms. The authors emphasized that the promising results of TRH in this setup suggest its potential for scale-up and industrial applications, particularly in treating textile wastewater. Future work should focus on large-scale implementation and optimization of this bio-adsorptive system for sustainable wastewater management.

**Figure 11:**
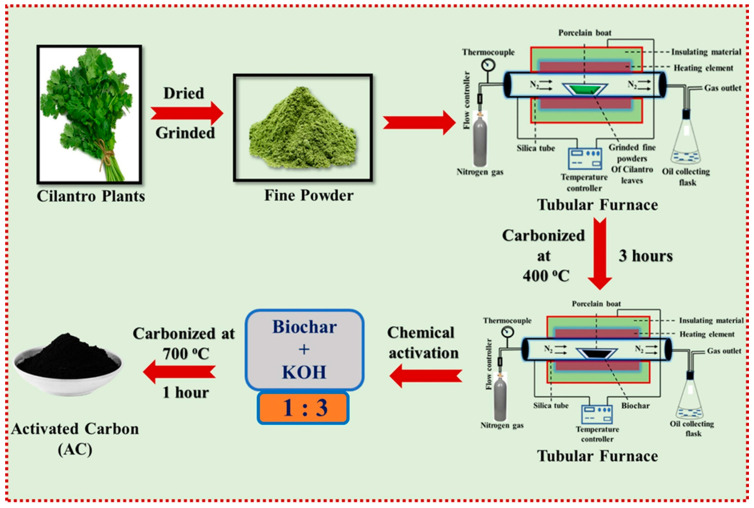
Schematic representation for the synthesis of AC by pyrolysis method. Reproduced with permission from [38].

Bhat et al. [38] conducted a study aimed at mitigating the global energy crisis by converting low-cost cilantro plants (*Coriandrum sativum*) into activated carbon (AC) with dual functionality—serving as an efficient adsorbent for toxic dye removal and as a high-performance material for supercapacitor applications. The process began with the collection of cilantro leaves and stems, which were chopped, dried, and ground into fine powder. The powdered biomass was then calcined at 400 °C for 3 h to obtain carbonaceous material, which was subsequently rinsed with double-distilled water (DDW) and dried in an oven at 70 °C for 24 h (Figure 11). FE-SEM images revealed that the resulting AC possessed a highly porous structure, indicating significant surface development suitable for adsorption and energy applications. Chemical activation was then performed using potassium hydroxide (KOH) at high concentrations, producing a robust material with an extensive network of interconnected pores and rough surface morphology. Adsorption studies demonstrated that the AC exhibited multilayer adsorption behavior characterized by heterogeneous active sites, as confirmed by the Freundlich isotherm model. Kinetic analyses showed that adsorption of Methyl Orange (MO) and Rhodamine 6G (Rh-6G) dyes followed the pseudo-second-order model, with high correlation coefficients (R^2^), signifying good agreement between experimental and theoretical data. Thermodynamic evaluations indicated that the adsorption process was spontaneous and feasible, as evidenced by negative ΔG° values. Positive ΔS° values suggested increased randomness at the solid–liquid interface during adsorption, while decreasing entropy with increasing dye concentration reflected greater molecular ordering as dyes adhered to the AC surface [79,80]. The material’s high porosity and abundant active sites were key factors contributing to its excellent adsorption efficiency and rapid dye removal capability. Overall, the study demonstrated that cilantro-derived activated carbon is a sustainable and cost-effective material with significant potential for industrial-scale applications in both environmental remediation and energy storage. Its unique structural and physicochemical properties make it a promising candidate for use as a superior adsorbent for organic pollutant removal and as an electrode material in supercapacitors.

Abid et al. [21] conducted a batch adsorption study to investigate the removal of malachite green (MG) dye from aqueous solutions using activated carbon prepared from walnut shells (ACWS). While zinc chloride (ZnCl_2_) has rarely been used for activating walnut shells, this study explored its effectiveness as a chemical activating agent in enhancing the adsorptive properties of the derived carbon material. The ZnCl_2_-activated carbon was synthesized using a walnut shell to ZnCl_2_ mass ratio of 0.5:3. ZnCl_2_ serves as an effective activating agent by promoting the development of an aromatic graphitic structure [81], thereby enriching the carbon content of the material. The activation mixture was washed repeatedly with distilled water until the pH stabilized at 6.2, followed by carbonization at 350 °C. The resulting ACWS exhibited a rough surface with numerous protrusions and a well-developed microporous structure featuring a range of pore sizes. Post-adsorption SEM analysis showed a smoother surface with fewer visible pores, suggesting that dye molecules had been adsorbed onto both the surface and internal pore network. The external morphology of the ACWS revealed many cavities of varying sizes and shapes, which act as entry points to interior micro- and mesopores—critical pathways for dye molecule diffusion. High-magnification imaging confirmed the abundance of small pores, many of which were capable of accommodating MG molecules. The average pore diameter was determined to be 16.33 nm, contributing to enhanced adsorption capacity due to increased surface area and active sites—key attributes of efficient porous adsorbents. Adsorption experiments were conducted at three different temperatures: 250, 350 and 550 °C. The results indicated that adsorption efficiency and removal capacity increased with temperature. At 350 °C, ACWS achieved a dye removal efficiency of 96% within one hour, while adsorption decreased to 68% at 250 °C. At 550 °C, the material completely changed into ash, suggesting over-carbonization. The improved performance at moderate temperatures (around 350 °C) was attributed to greater pore development and structural activation [82,83], consistent with earlier studies. Overall, the study demonstrated that ZnCl_2_ activation significantly enhances the structural and adsorptive properties of walnut shell–derived activated carbon. The findings highlight the importance of optimizing activation temperature to balance pore development and carbon stability, while also pointing to the need for further investigation into the adsorption kinetics and thermodynamics governing MG removal using ACWS.

Compared to commercial activated carbon (USD 10–20 kg^−1^), bio-derived adsorbents synthesized from agricultural and food wastes typically exhibit significantly lower production costs, ranging from USD 0.5–3 kg^−1^ depending on the precursor, activation method, and energy consumption. When normalized to dye removal performance, the estimated cost of dye removal using bio-derived carbons ranges from USD 0.02–0.10 per gram of dye removed, which is substantially lower than that of conventional activated carbon (USD 0.15–0.30 g^−1^). Additionally, the use of waste biomass eliminates raw material costs and reduces waste disposal expenses. Although chemical activation and high-temperature treatment increase production cost, these expenses are often offset by high adsorption capacity, regeneration ability, and extended service life. Therefore, bio-derived adsorbents demonstrate strong economic competitiveness and feasibility for large-scale wastewater treatment applications.

## 5. Dye Adsorption Performance of Bio-Derived Nanoparticles and Composites

**Figure 12:**
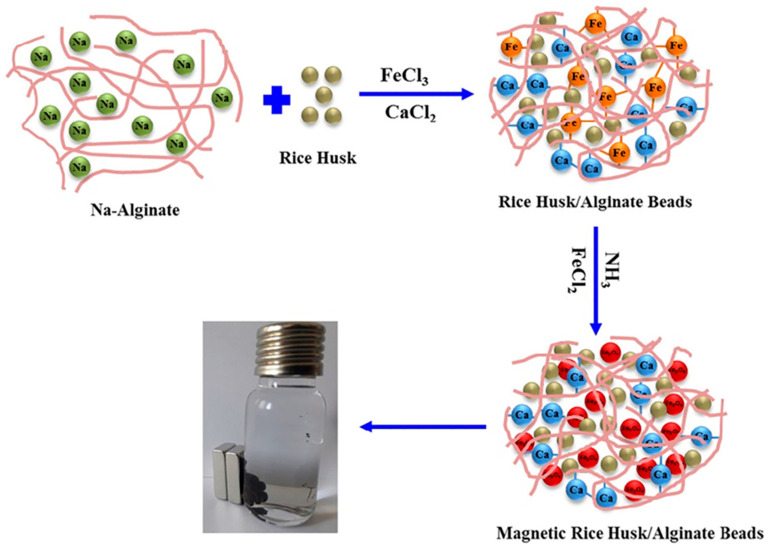
Schematic presentation of the preparation of the magnetic alginate/rice husk bio-composite beads. Reproduced with permission from [84].

Alver et al. [84] developed a novel magnetic alginate/rice husk (m-ALG/RH) bio-composite bead using the ionotropic gelation method and evaluated its efficiency for removing methylene blue (MB) dye from aqueous solutions (Figure 12). This research represents the first reported synthesis of magnetic alginate/rice husk composite beads designed for the adsorption of both organic and inorganic contaminants. In the preparation process, finely powdered rice husk (0.05 g, <45 μm) was dispersed in 10 mL of 2% (*w*/*v*) sodium alginate solution and stirred for 30 min. The resulting alginate/rice husk mixture was then dropped slowly through a syringe needle into a 50 mL solution containing 2% (*w*/*v*) CaCl_2_ and 0.05 M FeCl_3_ to form magnetic bio-composite beads. The introduction of rice husk enhanced the adsorption capacity of the beads due to the hydroxyl groups present on the silica surface, which interact synergistically with the carboxyl groups of the alginate chains, increasing the number of active adsorption sites. Characterization via SEM and EDX revealed that, after MB adsorption, the bead surfaces became smoother and exhibited increased carbon (C) and oxygen (O) peaks—confirming successful dye uptake on the bead surface. The magnetic alginate/rice husk beads demonstrated a high maximum adsorption capacity of 274.9 mg/g, indicating strong affinity toward MB molecules. Interestingly, the adsorption performances of the m-ALG/RH beads remained nearly constant within a pH range of 6–10, suggesting pH-independent adsorption behavior—an advantageous property for practical wastewater treatment applications. Thermodynamic analysis indicated that the adsorption process was spontaneous and favorable, as evidenced by negative ΔG° values, implying a high affinity between MB molecules and the bio-composite surface. The study established that magnetic alginate/rice husk bio-composite beads are an eco-friendly, cost-effective, and efficient adsorbent for dye removal from water. Their high adsorption capacity, chemical stability, and pH-insensitive performance make them promising candidates for scalable, sustainable water purification technologies.

Nausheen et al. [19] investigated the adsorption efficiency of Blue XGRRL dye removal using native clay, MnFe_2_O_4_/clay composite, and a biocomposite adsorbent. The study compared the performance of these materials in both batch and column adsorption systems to evaluate their potential for wastewater treatment applications. The MnFe_2_O_4_/clay composite was synthesized by thoroughly mixing 10 g of clay with equimolar amounts of MnCl_2_ and FeCl_3_, adjusting the pH to 10 using NH_4_OH, and stirring the mixture for 30 min. This was followed by heating at 95 °C for 2 h to promote the formation of the magnetic composite. The resulting MnFe_2_O_4_/clay material exhibited enhanced adsorptive characteristics compared to raw clay and the biocomposite. At pH 6, the adsorption behavior of the MnFe_2_O_4_/clay composite could not be completely explained by conventional mechanisms, suggesting that ion-exchange interactions may also contribute to dye removal. Among the tested adsorbents, the clay composite showed the highest adsorption capacity of 49.93 mg/g for Blue XGRRL dye. The adsorption data fitted well with the Langmuir isotherm model, indicating monolayer coverage on a homogeneous surface, and followed the pseudo-second-order kinetic model, suggesting chemisorption as the dominant process [85,86]. Thermodynamic analysis revealed that the adsorption of Blue XGRRL dye onto the MnFe_2_O_4_/clay composite was exothermic and spontaneous, with higher adsorption efficiency observed at lower temperatures. The decrease in dye removal at elevated temperatures was attributed to the weakening of adsorptive forces. Optimal adsorption was achieved under basic pH conditions, low adsorbent dosage, and reduced temperature. Column adsorption studies further demonstrated that the breakthrough time increased with greater bed height, indicating enhanced contact between dye molecules and the adsorbent due to reduced axial mass transfer dispersion and improved diffusion. Additionally, lower flow rates and higher dye concentrations were found to promote better dye sequestration. Overall, the MnFe_2_O_4_/clay composite exhibited superior adsorption efficiency compared to native clay and the biocomposite, confirming its promise as a low-cost, effective, and reusable adsorbent for removing Blue XGRRL dye from textile wastewater.

**Figure 13:**
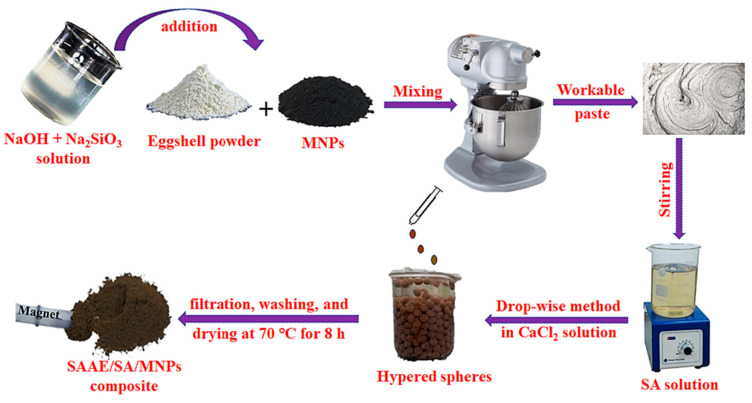
Flowchart illustrating the synthesis steps of SAAES/SA/MNPs adsorbent. Reproduced with permission from [18].

Elshimy et al. [18] developed a novel magnetic bio-based adsorbent (SAAES/SA/MNPs) using a composite of eggshell (ES) and magnetite nanoparticles (MNPs). The ES/MNPs mixture was first alkali-activated with a NaOH/Na_2_SiO_3_ solution, followed by impregnation with sodium alginate (SA) (Figure 13). The key innovation of the study was the use of eggshell waste as a raw material for producing an efficient adsorbent designed to remove organic pollutants—specifically crystal violet (CV) and methylene blue (MB) dyes—from aqueous environments. To prepare the material, a 2.0 wt.% SA solution was obtained by dissolving 2.0 g of SA powder in 100 mL of distilled water. This solution was then blended with the alkali-activated ES/MNPs mixture and stirred for 1 h at 50 °C. The resulting slurry was introduced dropwise into a 5 wt.% CaCl_2_ solution to form the SAAES/SA/MNPs beads. SEM micrographs revealed a rough, irregular surface morphology with diverse pores and cavities, indicating the formation of a microporous structure due to the agglomeration of SAAES/SA/MNPs particles bound by alginate crosslinking. TEM analysis further confirmed the presence of porous defects within the layered structure, likely enhancing the material’s dye adsorption performance [87]. The adsorption of MB and CV improved slightly within the pH range of 4.0–7.0, attributed to the deprotonation of active surface sites. The negative adsorption energy values (ΔE < 25 kJ/mol) suggested that physical adsorption forces dominated the dye–adsorbent interactions. Moreover, the bio-based adsorbent demonstrated excellent reusability, maintaining its dye removal efficiency after four regeneration cycles.

**Figure 14:**
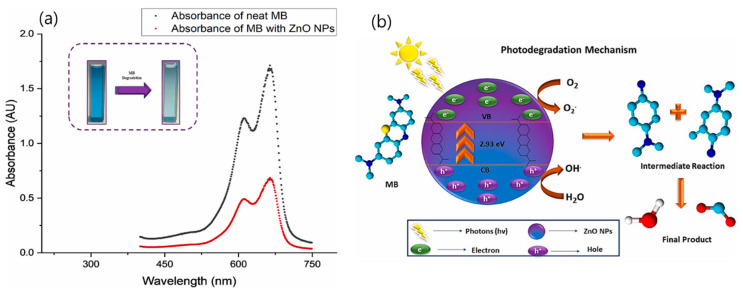
(**a**) Degradation of MB dye using ZnO NPs; and (**b**) Catalytic degradation mechanism of MB dye by ZnO NPs. Reproduced with permission from [88].

Jamil et al. [88] reported the synthesis of zinc oxide (ZnO) nanoparticles (NPs) through an eco-friendly biogenic route using an extract derived from the kernels of *Nigella sativa* (kalonji). The study also explored the utilization of *Punica granatum* (pomegranate) peels for cost-effective bio-oil production, employing the biosynthesized ZnO catalyst and comparing its performance with a conventional zeolite catalyst (ZSM-5). For nanoparticle synthesis, equal volumes (10 mL each) of plant extract and zinc nitrate solution [Zn(NO_3_)_2_] were mixed in a 100 mL beaker (1:1 ratio). The pH of the mixture was adjusted using 1 M NaOH, and the reaction was conducted at 90 °C for 120 min on a hot plate. SEM analysis revealed that the resulting ZnO nanoparticles exhibited diverse morphologies, predominantly consisting of nanorods and platelets that were well dispersed without aggregation. Photocatalytic degradation experiments demonstrated that approximately 71% of the dye was degraded within 60 min under UV irradiation (Figure 14a). The photocatalytic process involved the generation of electron–hole pairs, their subsequent separation, and the initiation of redox reactions on the nanoparticle surface, leading to the formation of highly reactive hydroxyl radicals (•OH) (Figure 14b). The conduction band electrons of ZnO interacted with molecular oxygen (O_2_), while holes in the valence band reacted with water molecules and hydroxide ions to produce •OH radicals. Because the reduction potential of H_2_O/OH and OH/OH is lower than the valence band potential of ZnO, efficient photocatalytic degradation occurred [89]. These findings confirmed the high photocatalytic efficiency of the biogenically synthesized ZnO nanoparticles.

Edal Queen et al. [15] successfully synthesized palladium nanoparticles (PdNPs) using an aqueous extract of cranberry fruit (ACE) through an optimized green synthesis approach. The resulting biogenic PdNPs demonstrated excellent catalytic efficiency for dye degradation, with optimization of parameters such as catalyst dosage, dye concentration, and environmental pH. Additionally, the synthesized PdNPs exhibited notable cytotoxic activity against the MCF-7 human breast cancer cell line, underscoring their multifunctional potential. For the synthesis, 10 mL of a 1 mM aqueous palladium(II) chloride (PdCl_2_) solution was mixed with 1 mL of cranberry extract. A visible color change in the solution signified nanoparticle formation, which was further confirmed by UV–Vis spectrophotometry. The photocatalytic performance of PdNPs increased with pH, reaching maximum degradation efficiencies of 96% for Sunset Yellow (SY) and 99% for Indigo Carmine (IC) at pH 9. The enhanced degradation at higher pH levels was attributed to the increased generation of hydroxyl radicals (•OH) by the photocatalyst, which effectively attacked the anionic dye molecules. Kinetic analysis revealed that the photocatalytic degradation of SY and IC followed a pseudo-first-order model, with correlation coefficients (R^2^) of 0.979 and 0.9835, respectively, indicating an excellent fit to the model. Structural characterization confirmed that the biogenic PdNPs possessed a face-centered cubic (FCC) crystalline structure, as verified by XRD. Morphological analyses via FE-SEM, EDS, and HR-TEM showed predominantly spherical nanoparticles (2–50 nm) with uniform dispersion and high stability. In addition to photocatalytic activity, the PdNPs demonstrated significant antibacterial efficacy against both Gram-positive and Gram-negative bacterial strains, and cytotoxicity against MCF-7 breast cancer cells, with an IC_50_ value of 68.88 mg/mL. Overall, the study highlights the potential of cranberry-mediated PdNPs as eco-friendly multifunctional nanomaterials for environmental remediation and biomedical applications.

**Figure 15:**
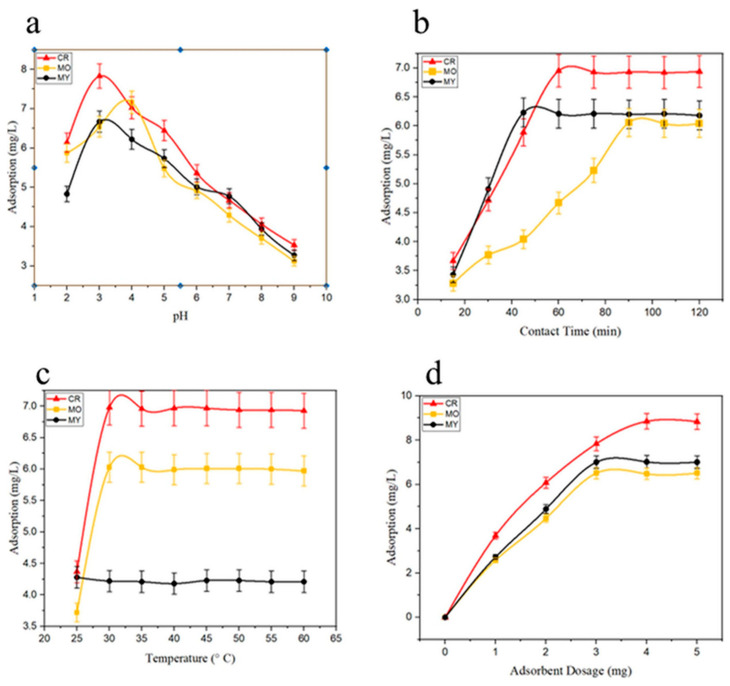
Various experimental set-ups for dye adsorption: (**a**) (pH: 2 to 9); (**b**) (contact time: 15 to 120 min); (**c**) (temperature: 25 to 60 °C); and (**d**) (adsorption dosage: 0 to 5 mg), with stirring speed (200 rpm). Reproduced with permission from [90].

Vijayasree et al. [90] developed a novel magnetic biosorbent (Cs@Fe_3_O_4_) composed entirely of natural components—chitosan and Fe^3+^ ions—derived from duck mouth clam shells and turkey berries, respectively. The bio-sorbent was synthesized via a co-precipitation method and applied for the removal of three anionic dyes: Congo Red (CR), Methyl Orange (MO), and Metanil Yellow (MY). For the synthesis of magnetic chitosan-coated Fe_3_O_4_ (Cs@Fe_3_O_4_), 2.45 g of FeCl_3_·6H_2_O was dissolved in HCl and mixed with 35 mL of turkey berry extract under stirring for 10 min. Subsequently, a 1% HCl solution containing 1 g of chitosan was added, followed by Na_2_SO_3_ to facilitate reduction. Ammonia and glutaraldehyde (25%) were then introduced as precipitating and cross-linking agents, respectively, yielding a black magnetic precipitate that was collected, dried, and used for adsorption studies. The adsorption experiments demonstrated that dye removal efficiency increased with higher adsorbent dosage due to the availability of more reactive surface sites. However, beyond a certain concentration, adsorption rates slowed, likely due to site saturation and possible particle–particle collisions at higher dosages [91]. Maximum dye removal efficiencies were recorded as 64.67% for Congo Red, 58.79% for Methyl Orange, and 53.33% for Metanil Yellow (Figure 15). The adsorption mechanism was primarily governed by electrostatic attraction between the negatively charged dye molecules and the protonated –NH_3_^+^ groups present on the adsorbent surface. Since no modification occurred on the –OH groups, the synthesized material was selective for anionic dyes, with limited affinity toward cationic species. Thermodynamic studies indicated that the adsorption process was both spontaneous and exothermic. Furthermore, the Cs@Fe_3_O_4_ adsorbent maintained stability and reusability across three adsorption–desorption cycles, confirming its potential as a sustainable and eco-friendly material for wastewater treatment applications.

Gupta et al. [92] investigated the use of magnetically modified *Aloe barbadensis Miller* leaves (MMABL) as an eco-friendly bio-sorbent for the removal of anionic dyes—Direct Blue 86 (DB-86) and Acid Yellow 36 (AY-36)—from aqueous solutions. The study focused on synthesizing, characterizing, and optimizing MMABL, along with evaluating its adsorption kinetics, equilibrium, thermodynamics, and underlying biosorption mechanisms. MMABL was prepared using a high-temperature co-precipitation method. An aqueous suspension of activated *A. barbadensis* leaf powder (100 mg/L, pretreated with HNO_3_) was mixed with 1 g of Fe_3_O_4_ nanoparticles in a 20 mL glass-stoppered bottle. The mixture was stirred at 120 rpm for 1 h at room temperature and then oven-dried at 90 °C for 5 h to obtain the MMABL powder. Surface characterization by SEM revealed that MMABL exhibited flake-like, porous, and cylindrical structures, attributed to the presence of Fe components. Comprehensive analyses using FTIR, BET, XRD, TGA, VSM, and EDX confirmed its high surface area, amorphous morphology, thermal stability, and superparamagnetic properties—key factors enhancing its adsorption performance. pH-dependent studies showed that dye adsorption was optimal at pH 2. At higher pH values, electrostatic repulsion between the negatively charged dye molecules and the deprotonated adsorbent surface led to reduced adsorption efficiency. Batch adsorption experiments demonstrated maximum adsorption capacities of 75.18 mg/g for AY-36 and 312 mg/g for DB-86. The adsorption process followed the Langmuir isotherm model (R^2^ > 0.99) and pseudo-second-order kinetics, indicating monolayer chemisorption. Thermodynamic analyses revealed Gibbs free energy changes (ΔG°) ranging from −17.50 to −20.45 kJ/mol for AY-36 and −18.20 to −21.54 kJ/mol for DB-86 between 293 and 323 K, suggesting a spontaneous process. Positive enthalpy (ΔH° = 3.22 kJ/mol for AY-36; 5.38 kJ/mol for DB-86) and entropy (ΔS° = 73.25 and 83.27 J·K^−1^·mol^−1^, respectively) values confirmed endothermic adsorption driven by increased randomness at the solid–liquid interface. Overall, MMABL proved to be an efficient, low-cost, and magnetically retrievable bio-sorbent with high affinity toward anionic dyes, offering significant promise for wastewater treatment. The authors also proposed future optimization of the process parameters using design of experiments (DoE) models. The adsorption performance of various bio-derived adsorbents and composites are listed in Table 1.

## 6. Conclusions and Future Perspectives

The review reveals that biomass-derived adsorbents—whether in raw, carbonized, or activated forms—offer a promising, low-cost, and eco-friendly solution for removing a broad range of anionic and cationic dyes. Their high surface area, abundant functional groups, and chemical tunability enable superior adsorption performance under optimized pH, temperature, and contact time conditions. Studies consistently show that adsorption onto these bio-based materials follows the Langmuir or Freundlich isotherm models and pseudo-second-order kinetics, indicating monolayer chemisorption or multilayer interactions. Moreover, their ability to be magnetically modified or composited with nanoparticles enhances separation, reusability, and overall process efficiency. Therefore, bio-derived carbon materials stand out as sustainable alternatives to conventional adsorbents for industrial-scale wastewater purification.

Despite the promising performance of bio-derived adsorbents, several critical bottlenecks must be addressed before large-scale industrial implementation. First, cost control during large-scale preparation remains challenging, particularly for chemically activated carbons that require high energy input and activating agents. Developing low-energy, solvent-free, or one-step activation strategies is essential. Second, adsorption selectivity in complex wastewater, especially high-salinity and multi-component industrial effluents, remains limited due to competitive ion effects. Third, regeneration efficiency and mechanical stability often decline after repeated cycles, reducing long-term applicability. Finally, the lack of standardized performance evaluation and techno-economic assessments hinders fair comparison and scale-up. Addressing these challenges through material engineering, process integration, and life-cycle analysis will be critical for transitioning bio-derived adsorbents from laboratory research to industrial wastewater treatment systems.

Future research should focus on improving the scalability, regeneration efficiency, and mechanical stability of bio-derived adsorbents to enable practical application in continuous-flow and industrial systems. Emphasis should also be placed on surface functionalization through nano-structuring, heteroatom doping, or hybridization with photocatalytic and magnetic materials to enhance selectivity and reusability. Integrating adsorption with advanced oxidation or biological processes could lead to synergistic dye degradation pathways. Additionally, techno-economic assessments, life-cycle analyses, and machine-learning-based optimization models can provide valuable insights for large-scale implementation. With continued innovation, bio-derived carbon adsorbents have the potential to redefine sustainable wastewater treatment technologies and contribute significantly to achieving global environmental and circular economy goals.

## Figures and Tables

**Table 1:** Adsorption performance of various bio-derived adsorbents and composites.

Adsorbent Material	Target Dye(s)	Adsorption Capacity (mg/g)	Removal Efficiency (%)	Key Parameters	Mechanism/Isotherm/Kinetics	Reference
Potato Peel (PP)	Acid Red 73	258.39	98.17	pH 2, 30 min equilibrium	Freundlich (R^2^ = 0.993), PSO, chemisorption	[58]
Trogoderma granarium biomass	Drimarine Yellow HF-3GL	481.9	78.7	pH 2, 30 °C	PSO, electrostatic attraction	[1]
Sesame Seed Cake	Methylene Blue	108.69	–	pH 3–9, 25 min	PSO, H-bonding, ANOVA-optimized	[37]
Date Seed Powder	Methyl Violet	59.5	–	100 °C drying, <300 µm	Langmuir, PSO, chemisorption	[52]
Walnut Shell (ZnCl_2_ activated)	Malachite Green	231.5	96 (at 350 °C)	350 °C optimum activation	Langmuir, temperature-dependent	[21]
Torrefied Rice Husk (TRH)	Methylene Blue	6.82	84	Inverse fluidized-bed, 0.00224 m/s	Enhanced mass transfer adsorption	[78]
Cilantro-derived Activated Carbon	Methyl Orange, Rhodamine 6G	467.29, 143.47	-	KOH activation	Freundlich, PSO, spontaneous adsorption	[38]
KOH-Activated Kiwi, Cucumber, Potato Peels	MB, MG, RhB	-	-	Multistage diffusion	Langmuir, PSO, ANN R^2^ = 0.996	[34]
TiO_2_–AC from Watermelon Rind	Congo Red, Phenol Red	70% ads. efficiency	-	Ultrasonic-assisted adsorption	Langmuir, monolayer adsorption	[11]
Magnetic Alginate/Rice Husk Beads	Methylene Blue	274.9	-	pH 6–10 stable	Langmuir, spontaneous, ΔG° < 0	[84]
MnFe_2_O_4_/Clay Composite	Blue XGRRL	49.93	-	pH 6	Ion exchange + adsorption	[19]
Raspberry-based Bio-carbon	Methylene Blue, Methyl Red	85–146; 70–103	-	Na_2_CO_3_ activation	Langmuir, PSO	[6]

## Data Availability

No new data were created or analyzed in this study. Data sharing is not applicable to this article.
